# Pfizer-BioNTech mRNA BNT162b2 Covid-19 vaccine protection against variants of concern after one versus two doses

**DOI:** 10.1093/jtm/taab083

**Published:** 2021-05-28

**Authors:** Laith J Abu-Raddad, Hiam Chemaitelly, Hadi M Yassine, Fatiha M Benslimane, Hebah A Al Khatib, Patrick Tang, Joel A Malek, Peter Coyle, Houssein H Ayoub, Zaina Al Kanaani, Einas Al Kuwari, Andrew Jeremijenko, Anvar Hassan Kaleeckal, Ali Nizar Latif, Riyazuddin Mohammad Shaik, Hanan F Abdul Rahim, Gheyath K Nasrallah, Mohamed Ghaith Al Kuwari, Hamad Eid Al Romaihi, Mohamed H Al-Thani, Abdullatif Al Khal, Adeel A Butt, Roberto Bertollini

**Affiliations:** Infectious Disease Epidemiology Group, Weill Cornell Medicine-Qatar, Cornell University, Doha, Qatar; World Health Organization Collaborating Centre for Disease Epidemiology Analytics on HIV/AIDS, Sexually Transmitted Infections, and Viral Hepatitis, Weill Cornell Medicine–Qatar, Cornell University, Qatar Foundation – Education City, Doha, Qatar; Department of Population Health Sciences, Weill Cornell Medicine, Cornell University, New York, NY, USA; Infectious Disease Epidemiology Group, Weill Cornell Medicine-Qatar, Cornell University, Doha, Qatar; World Health Organization Collaborating Centre for Disease Epidemiology Analytics on HIV/AIDS, Sexually Transmitted Infections, and Viral Hepatitis, Weill Cornell Medicine–Qatar, Cornell University, Qatar Foundation – Education City, Doha, Qatar; Biomedical Research Center, Member of QU Health, Qatar University, Doha, Qatar; Department of Biomedical Science, College of Health Sciences, Member of QU Health, Qatar University, Doha, Qatar; Biomedical Research Center, Member of QU Health, Qatar University, Doha, Qatar; Department of Biomedical Science, College of Health Sciences, Member of QU Health, Qatar University, Doha, Qatar; Biomedical Research Center, Member of QU Health, Qatar University, Doha, Qatar; Department of Biomedical Science, College of Health Sciences, Member of QU Health, Qatar University, Doha, Qatar; Department of Pathology, Sidra Medicine, Doha, Qatar; Genomics Laboratory, Weill Cornell Medicine-Qatar, Cornell University, Doha, Qatar; Department of Genetic Medicine, Weill Cornell Medicine-Qatar, Cornell University, Doha, Qatar; Biomedical Research Center, Member of QU Health, Qatar University, Doha, Qatar; Hamad Medical Corporation, Doha, Qatar; Wellcome-Wolfson Institute for Experimental Medicine, Queens University, Belfast, UK; Department of Mathematics, Statistics, and Physics, Qatar University, Doha, Qatar; Hamad Medical Corporation, Doha, Qatar; Hamad Medical Corporation, Doha, Qatar; Hamad Medical Corporation, Doha, Qatar; Hamad Medical Corporation, Doha, Qatar; Hamad Medical Corporation, Doha, Qatar; Hamad Medical Corporation, Doha, Qatar; College of Health Sciences, QU Health, Qatar University, Doha, Qatar; Genomics Laboratory, Weill Cornell Medicine-Qatar, Cornell University, Doha, Qatar; Department of Genetic Medicine, Weill Cornell Medicine-Qatar, Cornell University, Doha, Qatar; Primary Health Care Corporation, Doha, Qatar; Ministry of Public Health, Doha, Qatar; Ministry of Public Health, Doha, Qatar; Hamad Medical Corporation, Doha, Qatar; Department of Population Health Sciences, Weill Cornell Medicine, Cornell University, New York, NY, USA; Hamad Medical Corporation, Doha, Qatar; Ministry of Public Health, Doha, Qatar

**Keywords:** SARS-CoV-2, alpha variant, beta variant, delta variant, B1.617.2, B1.1.7

##  

Strategies for rolling out vaccination against Coronavirus Disease 2019 (Covid-19) varied across countries. A key question is whether delaying administration of the second vaccine dose to vaccinate the largest number of people in the shortest time, in situations of limited vaccine supplies and high incidence, could avert more disease cases, hospitalizations and deaths than the current protocol of a second dose shortly after the first dose.

**Figure 1:**
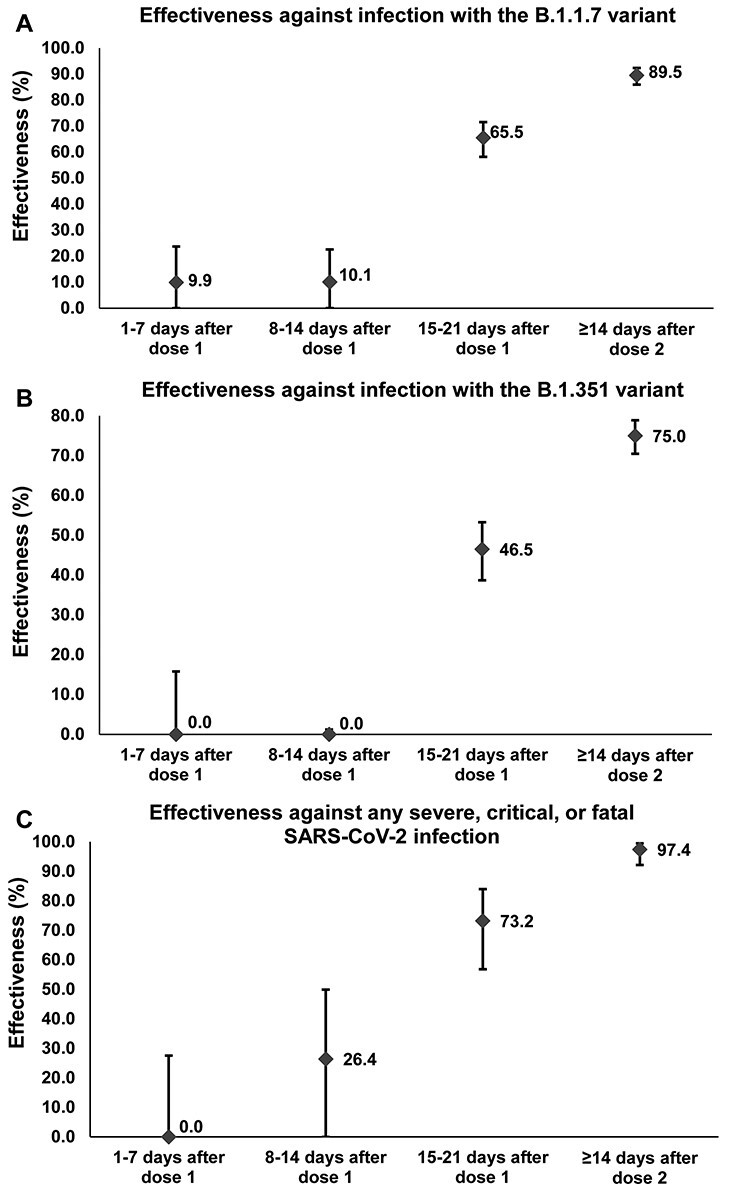
The messenger RNA vaccine BNT162b2 (Pfizer-BioNTech) effectiveness against infection and against disease in the weeks following the first dose. Error bars indicate the 95% confidence intervals for vaccine effectiveness estimates.

**Table 1:** The messenger RNA vaccine BNT162b2 (Pfizer-BioNTech) effectiveness against infection and against disease in the weeks following vaccination with the first dose

	**1–7 days after dose 1**					**8–14 days after dose 1**				
	**Cases (PCR positive)**		**Controls (PCR negative)**		**Effectiveness in % (95% CI)** ^**a**^	**Cases (PCR positive)**		**Controls (PCR negative)**		**Effectiveness in % (95% CI)** ^**a**^
	**Vaccinated**	**Unvaccinated**	**Vaccinated**	**Unvaccinated**		**Vaccinated**	**Unvaccinated**	**Vaccinated**	**Unvaccinated**	
**Effectiveness against infection**										
Any infection with the B.1.1.7 variant^b^	279	17 262	309	17 232	9.9 (0.0–23.7)	346	17 331	384	17 293	10.1 (0.0–22.6)
Any infection with the B.1.351 variant^c^	276	19 071	276	19 071	0.0 (0.0–15.8)	526	19 247	470	19 303	0.0 (0.0–1.2)
**Effectiveness against disease**										
Any severe, critical, or fatal disease with the B.1.1.7 variant^d^	12	431	19	424	37.9 (0.0–72.8)	9	426	14	421	36.5 (0.0–76.0)
Any severe, critical, or fatal disease with the B.1.351 variant^e^	16	321	6	331	0.0 (0.0–0.0)	17	322	6	333	0.0 (0.0–0.0)
Any severe, critical, or fatal disease with any SARS-CoV-2 infection^f^	49	1807	44	1812	0.0 (0.0–27.6)	52	1831	70	1813	26.4 (0.0–49.9)
	**15–21 days after dose 1**					**≥14 days after second dose**				
	**Cases (PCR positive)**		**Controls (PCR negative)**		**Effectiveness in % (95% CI)** ^**a**^	**Cases (PCR positive)**		**Controls (PCR negative)**		**Effectiveness in % (95% CI)** ^**a**^
	**Vaccinated**	**Unvaccinated**	**Vaccinated**	**Unvaccinated**		**Vaccinated**	**Unvaccinated**	**Vaccinated**	**Unvaccinated**	
**Effectiveness against infection**										
Any infection with the B.1.1.7 variant^b^	148	17 380	422	17 106	65.5 (58.2–71.5)	50	16 354	465	15 939	89.5 (85.9–92.3)
Any infection with the B.1.351 variant^c^	338	19 400	623	19 115	46.5 (38.7–53.3)	179	19 396	698	18 877	75.0 (70.5–78.9)
**Effectiveness against disease**										
Any severe, critical, or fatal disease with the B.1.1.7 variant^d^	7	434	24	417	72.0 (32.0–90.0)	0	401	20	381	100.0 (81.7–100.0)
Any severe, critical, or fatal disease with the B.1.351 variant^e^	9	336	20	325	56.5 (0.0–82.8)	0	300	14	286	100.0 (73.7–100.0)
Any severe, critical, or fatal disease with any SARS-CoV-2 infection^f^	23	1845	83	1785	73.2 (56.8–84.0)	3	1692	109	1586	97.4 (92.2–99.5)

^a^Vaccine effectiveness was estimated using the test-negative, case–control study design.^2^ Cases and controls were matched one-to-one by age, sex, nationality, and reason for PCR testing. Vaccine effectiveness is given by^2^  $\mathrm{Vaccine}\ \mathrm{effectiveness}=1-\frac{\mathrm{vaccinated}\ \mathrm{among}\ \mathrm{cases}\times \mathrm{unvaccinated}\ \mathrm{among}\ \mathrm{controls}}{\mathrm{vaccinated}\ \mathrm{among}\ \mathrm{controls}\times \mathrm{unvaccinated}\ \mathrm{among}\ \mathrm{cases}}$.

^b^Any B.1.1.7 PCR-confirmed infection. A B.1.1.7 infection is proxied as an S-gene ‘target failure’ case using the TaqPath COVID-19 Combo Kit platform (Thermo Fisher Scientific, USA), applying the criterion of PCR cycle threshold value ≤ 30 for both the N and ORF1ab genes, but a negative outcome for the S-gene. The median date of vaccination was March 1 2021 for the cases and February 28 2021 for their matched controls.

^c^Any B.1.351 PCR-confirmed infection. With only B.1.351 and B.1.1.7 cases identified in the viral genome sequencing after 7 March 2021, a B.1.351 infection is proxied as the complement of the B.1.1.7 criterion, that is any infection with a Ct value ≤ 30 for the N, ORF1ab and S genes between March 8 and 31 2021. The median date of vaccination was March 7 2021 for the cases and March 1 2021 for their matched controls.

^d^Any B.1.1.7 PCR-confirmed infection that led to severe, critical or fatal disease. Severe disease, critical disease and COVID-19 death were defined based on the World Health Organization criteria for classifying SARS-CoV-2 infection severity^3^ and COVID-19-related death.^4^

^e^Any B.1.351 PCR-confirmed infection that led to severe, critical or fatal disease. Severe disease, critical disease and COVID-19 death were defined based on the World Health Organization criteria for classifying SARS-CoV-2 infection severity^3^ and COVID-19-related death.^4^

^f^Any PCR-confirmed infection that led to severe, critical or fatal disease. With the dominance of both B.1.1.7 and B.1.351 variants during the study period, this effectiveness is a combined measure against both of these variants. Severe disease, critical disease and COVID-19 death were defined based on the World Health Organization criteria for classifying SARS-CoV-2 infection severity^3^ and COVID-19-related death.^4^

BNT162b2 (Pfizer-BioNTech) vaccine effectiveness against the severe acute respiratory syndrome coronavirus 2 (SARS-CoV-2) in Qatar was recently reported with focus on individuals who completed 14 days after the second dose.^1^ Here, we provide a follow-up analysis of how vaccine protection develops week-by-week after the first dose.

Data for SARS-CoV-2 were extracted from Qatar’s nationwide digital-health information platform. The platform hosts the national centralized SARS-CoV-2 databases that captured all vaccination records, polymerase chain reaction (PCR) testing, and COVID-19 hospitalizations and deaths since epidemic start.^1^ The study was conducted from 1 February to 31 March 2021, the period of rapid mass vaccination scale-up. Vaccine effectiveness was estimated using the test-negative case–control study design.^2^ Cases and controls were matched one-to-one by age, sex, nationality and reason for PCR testing. Effectiveness was estimated against documented infection with the B.1.1.7 or B.1.351 variants, as well as against severe, critical or fatal disease due to any SARS-CoV-2 infection. Classification of COVID-19 case severity (acute-care hospitalizations),^3^ criticality (ICU hospitalizations)^3^ and fatality^4^ followed the World Health Organization guidelines. Further details on study methods can be found in our previous publication.^1^

Between 1 February and 31 March 2021, 333 764 individuals received at least one BNT162b2 vaccine dose, of whom 250 619 completed two doses. Two-thirds (60.8%) of those vaccinated were men, and the median age was 40 years. Median time elapsed between the first and second doses was 21 days, and 98.4% of individuals received their second dose ≤ 25 days after the first dose. Effectiveness against infection with B.1.1.7 or B.1.351 was negligible for 2 weeks after the first dose (Figure 1). Effectiveness increased rapidly during the third week to 65.5% (95% CI: 58.2–71.5) against B.1.1.7 and 46.5% (95% CI: 38.7–53.3) against B.1.351 (Table 1). Eventually, ≥14 days after the second dose, effectiveness reached 89.5% (95% CI: 85.9–92.3) against B.1.1.7 and 75.0% (95% CI: 70.5–78.9) against B.1.351.

Effectiveness against severe, critical or fatal disease (predominantly due to B.1.1.7 and B.1.351^5^) was negligible during the first week, reached 26.4% (95% CI: 0.0–49.9) in the second week, and grew to 73.2% (95% CI: 56.8–84.0) in the third week (Table 1). Eventually, ≥14 days after the second dose, effectiveness reached 97.4% (95% CI: 92.2–99.5).

Development of protection against infection and disease accelerated in the third week after the first dose, right before the second dose, reaching nearly 75% of the value attained ≥14 days after the second dose. Protection increased most rapidly against hospitalization and death and slowest against B.1.351 infection. While protection of one dose beyond 21 days could not be assessed, and existing protocol requires a second dose for optimal protection, these findings support the strategy of delaying the second dose to vaccinate the largest number of people in the shortest time, in situations of limited vaccine supplies and high incidence, given the substantial protection achieved after only one dose. In areas where B.1.351 is at high incidence, delivering the second vaccine dose at 3–6 weeks after the first dose may be considered with the lower and slower build-up of protection against this variant.

**Key Points**

- This population-based study documents BNT162b2 vaccine protection week-by-week after the first dose.
- 75% of protection against infection and disease is reached 15–21 days after the first dose.
- Protection increased most rapidly against hospitalization and death and slowest against B.1.351 infection.
- While protection of one dose beyond 21 days could not be assessed, findings support delaying the second vaccine dose in situations of limited vaccine supplies and high incidence.

## Authors’ contributions

L.J.A. co-conceived and co-designed the study, led the statistical analyses, and co-wrote the first draft of the article. A.B. and R.B. co-conceived and co-designed the study. H.C. co-designed the study, performed the statistical analyses and co-wrote the first draft of the article. All authors contributed to data collection and acquisition, database development, discussion and interpretation of the results, and to the writing of the manuscript. All authors have read and approved the final manuscript.
